# Response to: ‘A dose–response relationship between severity of disc degeneration and intervertebral disc height in the lumbosacral spine’

**DOI:** 10.1186/s13075-016-0944-y

**Published:** 2016-02-08

**Authors:** Kaj S. Emanuel, Idsart Kingma, Marco N. Helder, Theodoor H. Smit

**Affiliations:** Department of Orthopaedic Surgery, VU University Medical Center, MOVE Research Institute Amsterdam, De Boelenlaan 1118, 1081 HZ Amsterdam, The Netherlands; Department of Human Movement Sciences, Faculty of Behavioural and Movement Sciences, Vrije Universiteit Amsterdam, MOVE Research Institute Amsterdam, Van Boechhorststraat 7, 1081 BT Amsterdam, The Netherlands

With much interest we read the recent paper by Teichtahl et al. [1], who gave novel insight into the association between disc degeneration and intervertebral disc height. The current golden standard, the Pfirrmann Score, has a good internal validity. However, its subjective nature, low discriminative power, and moderate relation to physiological and clinical parameters have inspired many investigators to search for a better alternative. We support this enthusiastically, as there is a great need for a measure with higher clinical relevance.

Loss of water is a feature of disc degeneration. Therefore, disc height or volume is a simple parameter to monitor discal changes in large groups. The data shown in this research, however, suggest that this measure is probably not a clinically relevant parameter. Figure 2b of Teichtahl et al. [1] shows that an individual with a corrected disc height of 11 mm easily fits within one standard deviation of the averages found for Pfirrmann grades 2, 3, and 4, leaving no option to discriminate between mild-to-severe degeneration. Moreover, it is important to recognize that disc height is a temporary state of the discus, strongly dependent on loading history [2] and therefore highly variable within subjects. Over 20 % average disc height variation within the same day has been reported [3], comparable with two Pfirrmann grades on a single day. The high inter-subject and intra-subject variation emphasizes the limitations for using this measure as a relevant parameter, both in epidemiological studies and in clinical practice. This may explain why Videman et al. [4] found that disc height explained only 7 % of the variance in low-back pain.

To improve the current golden standard, the relation to physiological parameters can be used as a benchmark. We would like to highlight that several continuous magnetic resonance imaging measures have already been developed, some of which have shown encouraging relations to biomechanical properties and biochemical content, in addition to significant associations with the Pfirrmann Score. Examples of these measures are T1ρ [5], T2* mapping, and quantitative T2 mapping (e.g., [6]). Evaluating new measures in this way is a promising development, because only with stable and relevant measures can the relation between disc degeneration and low-back pain be investigated properly.

In conclusion, there is a great need for relevant measures of disc degeneration. However, the results of this study indicate that disc height should not be used as a singular measure for disc degeneration.
